# Numerical Analysis of Combustion Characteristics in an Industrial Float Glass Furnace: Effects of Burner Inclination and Excess Air Ratio

**DOI:** 10.3390/ma19143094

**Published:** 2026-07-18

**Authors:** Yuqin Liu, Hao Feng, Liming Zou, Xiaocheng Liang, Qingyue Chen, Benjun Cheng

**Affiliations:** 1 School of Energy Science and Engineering, Central South University, Changsha 410083, China; 13228134442@163.com (Y.L.); 253911025@csu.edu.cn (L.Z.); cqyupup@csu.edu.cn (Q.C.); 2 CRRC Zhuzhou Institute Co., Ltd., Zhuzhou 412005, China; 15675191585@163.com; 3 School of Materials Science and Engineering, Shanghai University, Shanghai 200444, China; lxc2025@shu.edu.cn; 4 State Key Laboratory of Advanced Refractories, Shanghai University, Shanghai 200444, China

**Keywords:** float glass furnace, dual-cooler structure, combustion-space simulation, burner inclination angle, excess air ratio

## Abstract

**Highlights:**

- A validated model clarifies combustion features of a 1300 t/d float glass furnace.
- Lower burner inclination widens the high-temperature flame zone.
- Excess air ratio 1.15 balances completeness, uniformity, and flue-gas heat loss.

**Abstract:**

Clarifying combustion-space behavior is essential for operating large-tonnage natural gas-fired float glass furnaces with complex single-furnace dual-cooler layouts. In this study, a three-dimensional computational fluid dynamics model of the gas-phase combustion space of a 1300 t/d natural gas-fired float glass furnace was developed and validated using crown-temperature measurements, with a maximum relative error of 3.3%. The effects of burner inclination angle (β = 5°, 10°, and 15°) and excess air ratio (α = 1.0–1.20) on temperature distribution, flame morphology, and flue-gas recirculation were investigated. The results show that β = 5° produces a more horizontally extended natural-gas jet, enhances contact with preheated air, and forms a wider high-temperature region, with a maximum temperature of 2512 K. Increasing the excess air ratio improves combustion completeness and enlarges the high-temperature region; however, further increasing α from 1.15 to 1.20 provides only marginal thermal benefits while increasing sensible heat loss through the exhaust gas. Among the investigated operating conditions, β = 5° and α = 1.15 achieve the lowest outlet flue-gas specific enthalpy of 755 KJ/Kg.

## 1. Introduction

As a basic material with excellent light transmittance, mechanical strength, and chemical stability, glass plays an irreplaceable role in fields such as construction, aerospace, automotive manufacturing and photovoltaics. Glass compositions are complex and diverse. In addition to soda-lime glass, which is mainly composed of Na_2_SiO_3_ and CaSiO_3_, other types include tempered glass, colored glass, potassium glass, and glass-ceramics [1,2,3,4,5]. Recent studies have also shown that glass-derived materials can be tailored as glass-powder-modified cementitious materials, slag-based glass-ceramics, radiation-shielding bismuth borate glasses, and colored glass for building-integrated photovoltaics [6,7,8,9]. Based on differences in heating energy sources, process characteristics, and application scenarios, mainstream glass melting methods can be divided into flame melting, electric melting, and plasma melting. Among these, the electric melting method can greatly improve energy efficiency and reduce pollution emissions [10,11], but it has high energy consumption costs and is generally used for small-batch glass production. The plasma melting method offers the advantage of rapid heating and can be used for sintering refractory metals and ceramics [12]. However, among the three methods, the most widely used glass melting method is flame melting. The process that implements this melting is the float glass process, and the primary equipment is the float glass melting furnace. This process utilizes the tension between molten glass and molten metal to spread the molten glass on the surface of the molten metal [13]. During this process, the molten glass forms a continuous glass ribbon on the molten metal bath, and its thickness and surface flatness are controlled by the stretching and cooling processes before annealing.

With increasingly strict requirements for glass quality, energy saving, and emission reduction, large-tonnage natural gas-fired float glass furnaces have been developed and applied in industrial production. The 1000-ton class float glass-melting furnace with a single-furnace single-cooler can increase production, but the quality and variety of the produced glass have not improved. Therefore, during this process, the structure of large-tonnage glass melting furnaces has been upgraded to natural gas-fired float glass melting furnaces of a single-furnace dual-cooler. In industrial float glass production, the combustion performance of regenerative furnaces plays a decisive role in determining fuel consumption, thermal efficiency, glass melting quality, refractory service life, and pollutant emissions [14]. Due to the short development cycle, the temperature distribution patterns within the combustion space of the furnace and the influence of combustion heat on the melting and flow behavior of the glass melt remain unclear. Moreover, due to the increase in the number of branch lines and ports, the heat load of the terminal ports will increase. The ratio of combustion fuel to air lacks specific theoretical guidance, which can lead to uneven temperature distribution in the combustion space and, consequently, uneven temperature distribution on the glass melt surface, affecting glass quality. Therefore, it is necessary to conduct in-depth research on the temperature distribution and flow patterns within the combustion space. The combustion and heat-transfer processes in a glass melting furnace involve turbulent flow, fuel oxidation, high-temperature radiation, wall heat loss, and coupling with the glass-melt surface. Direct industrial measurement inside the furnace is difficult because of the high temperature, enclosed space, and harsh operating environment. Computational fluid dynamics (CFD) provides an effective method for analyzing the temperature field, flow field, and flame structure in the combustion space, and it can provide useful guidance for burner arrangement and operating-parameter adjustment.

In the early stages of numerical combustion simulation, scholars mainly used simplified models and computer programs. To verify the reliability of numerical simulation methods for simulating float glass melting furnaces, researchers [15,16,17] established a fluid dynamics mathematical model of the glass melting furnace and studied flame and heat transfer processes through numerical simulation, demonstrating that numerical simulation can, to a certain extent, guide the float glass production process. Yu et al. [18] constructed and tested a 1:15 cold-flow scale model of a 500 t/d oil-fired glass melting furnace and studied the influence of three different burner positions on the combustion aerodynamics and flame geometry inside the furnace. Han et al. [19] established an oxy-fuel combustion glass melting furnace model, focusing on exploring the influence of the oxygen content in the combustion air on the flow-field and temperature-field distribution patterns of the flame in the combustion space. The results showed that increasing the oxygen content helps accelerate the combustion rate under otherwise identical conditions. Internationally, in their early studies, R.L. Curran et al. [20,21,22] focused on fluid flow and heat transfer problems inside the furnace and were committed to optimizing the calculation time for the entire glass melting furnace simulation [23]. A. Ungan et al. [24] introduced the mathematical formulas and numerical solution procedures for simulating the transfer process in the glass melting tank and verified the practicality of the coupling between models [25]. Regarding the innovation and improvement of combustion models, the Eddy Dissipation Model (EDM) [26] and Eddy Dissipation Concept (EDC) Model [27] proposed by B. Magnussen et al. consider the intermittent occurrence of reactants in turbulent flames and correlate the combustion rate with the dissipation rate of eddies, providing an effective tool for analyzing the turbulent combustion process in glass melting furnaces. Subsequently, the proposal of the Steady Laminar Flamelet Model (SFM) [28] based on mixture fraction and the Flamelet Generated Manifold (FGM) method [29] further incorporated the interaction between steady laminar flow, turbulence, and chemistry, significantly enhancing the completeness of combustion simulation for glass melting furnaces.

Based on this, researchers have conducted extensive exploration on fluid dynamics and heat transfer modeling in glass furnaces and achieved significant progress [30]. For example, Pilon et al. [31,32] completed the construction of a glass fluid circulation model for the glass melting furnace and controlled the flow field and temperature field of the glass flow by adjusting the combustion in the combustion space. Dzyuzer and Shvydki [15] developed a numerical model combining the fluid dynamics of the melting tank with external heat transfer and also provided calculation results for the temperature and flow fields of the glass melting furnace. The adequacy of the calculation results under actual operating conditions was verified. These studies show that the distribution of heat release and surface heat flux strongly affects the thermal state and circulation of molten glass. Nevertheless, most existing studies focus on conventional furnace structures or general heat-transfer mechanisms. The parameter–structure coupling effect in large-tonnage natural gas-fired float glass furnaces with a single-furnace dual-cooler structure has not been sufficiently discussed, especially with respect to burner inclination angle and excess air ratio.

Hydrogen has recently attracted increasing attention as a promising low-carbon fuel for high-temperature industrial furnaces owing to its potential for reducing direct CO_2_ emissions during combustion. However, compared with natural gas, the application of hydrogen in existing industrial glass furnaces still faces several technical and practical challenges. Owing to its higher adiabatic flame temperature and faster flame propagation characteristics, hydrogen combustion may promote thermal NOₓ formation under conventional burner configurations and often requires modifications to burner design and combustion strategies to ensure stable and safe operation. Moreover, the low volumetric energy density of hydrogen necessitates substantial changes to fuel supply systems, storage facilities, and safety management. Consequently, natural gas currently remains the predominant fuel used in commercial float glass production. Therefore, the present study focuses on the combustion optimization of an industrial natural-gas-fired float glass furnace under current industrial operating conditions [33,34,35].

Although CFD has been widely employed to investigate combustion and heat transfer in glass furnaces, most previous studies have focused on laboratory-scale furnaces, simplified geometries, or single operating parameters. Systematic investigations of large-scale industrial float glass furnaces under practical operating conditions remain limited. In particular, the influence of burner inclination angle and excess air ratio on flame development, flow characteristics, and thermal performance has not been comprehensively clarified. Furthermore, quantitative evaluations of combustion optimization for industrial natural-gas-fired float glass furnaces are still insufficient, restricting the practical application of numerical simulation to furnace operation optimization. Therefore, this study establishes a three-dimensional CFD model of the combustion space of a 1300 t/d natural-gas-fired single-furnace dual-cooler float glass furnace to optimize its combustion characteristics under current industrial operating conditions. The objective is not to perform global mathematical optimization, but to clarify how burner inclination angle and excess air ratio affect flame morphology, temperature distribution, and flue gas recirculation in this special furnace configuration. The main contribution of this work is to reveal the relative influence of these two operating parameters on combustion-space flow and thermal behavior, thereby providing a reference for parameter adjustment in similar industrial furnaces.

## 2. Model Establishment and Grid Independence Verification

This section describes the establishment of the three-dimensional CFD model for the industrial float glass furnace. The boundary conditions and mesh generation procedure are presented in detail. Furthermore, a grid independence verification is performed to ensure that the numerical results are insensitive to mesh resolution and provide a reliable basis for the subsequent numerical simulations.

### 2.1. Structural Dimensions of the Model

**Figure 1:**
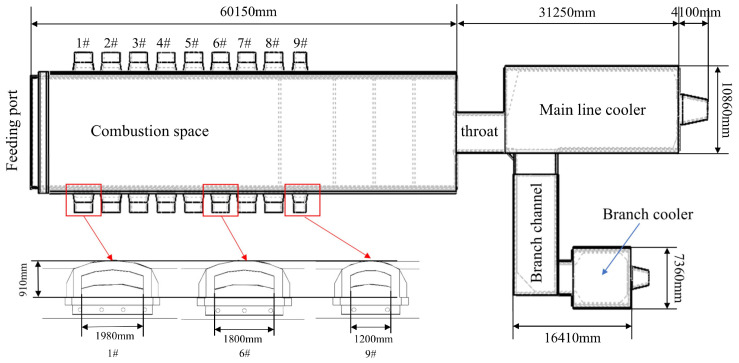
Main structural dimensions of the melting furnace (top view).

**Figure 2:**
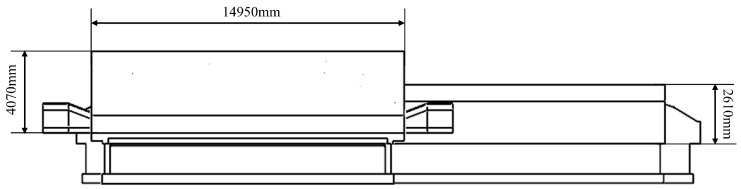
Main structural dimensions of the melting furnace (left view).

**Figure 3:**
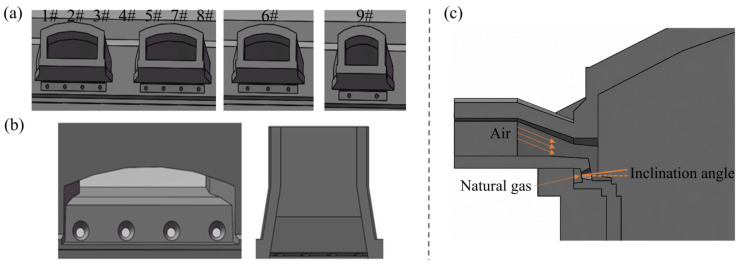
(**a**) Burner installation positions under the nine ports; (**b**) partial enlarged view of the outlet port; (**c**) schematic of the burner installation angle.

A three-dimensional physical model of a 1300 t/d natural-gas-fired float glass melting furnace with a single-furnace dual-cooler configuration was established using SolidWorks 2024. The main structural dimensions of the model in the top view and left view are presented in Figure 1 and Figure 2, respectively. To further illustrate the installation configuration of the natural gas burners, Figure 3 shows the burner installation positions beneath the nine ports, a partial enlarged view of the outlet port, and a schematic of the burner inclination angle.

### 2.2. Boundary Settings

The air and fuel inlets were both defined as mass-flow inlets. The O_2_ content in the air was set to 21%, and the air was preheated to 665 K by the regenerative system. The fuel temperature was 300 K. The air flow rate at the nine air inlets and the fuel flow rate at the burner nozzles were calculated and specified according to the fuel distribution ratios listed in Table 1. The flue gas outlet was defined as a pressure outlet with a gauge pressure of 0 Pa and a backflow temperature of 1350 K. The outer walls of the glass-melting furnace were subjected to convective heat transfer with the surrounding atmosphere at 320 K, with an overall heat transfer coefficient of 5 W/(m^2^·K). This value was used as an engineering heat-loss boundary for the large external furnace wall under natural-convection conditions. The emissivity for radiative heat transfer from the flue gas to the inner furnace walls was set to 0.6, and the emissivity for radiative heat transfer from the flue gas to the glass melt surface was set to 0.75. The main boundary condition descriptions are summarized in Table 2 below.

The turbulence intensity and hydraulic diameter at the inlets and outlets were determined by using Equations (1) and (2).

(1) $$I=\frac{u^{\prime }}{u_{avg}}=0.16{\left(Re_{D_{H}}\right)}^{-\frac{1}{8}}$$

(2) $$D_{H}=4\frac{A}{P}$$

where $u^{\prime }$ represents the root mean square of the turbulent fluctuating velocity, $u_{avg}$ is the average velocity, and $Re_{D_{H}}$ represents the Reynolds number with the hydraulic diameter as the characteristic length. A represents the fluid cross-sectional area, and P represents the fluid cross-sectional perimeter.

### 2.3. Material Physical Parameters

The computational domain in this study consists of the gas-phase combustion space and the refractory solid wall domain. The gas-phase domain includes natural gas, preheated air, and combustion products, while the solid domain represents the refractory bricks of the furnace structure. The glass-melt surface is treated as a stationary thermal boundary with a specified radiative property. Therefore, the present model should be regarded as a combustion-space model. The fluid region primarily consists of natural gas (composed of CH_4_ and C_2_H_6_), combustion air, and combustion products (CO_2_ and H_2_O). The physical parameters of the main materials and gas components are shown in Table 3.

### 2.4. Grid Independence Verification

**Figure 4:**
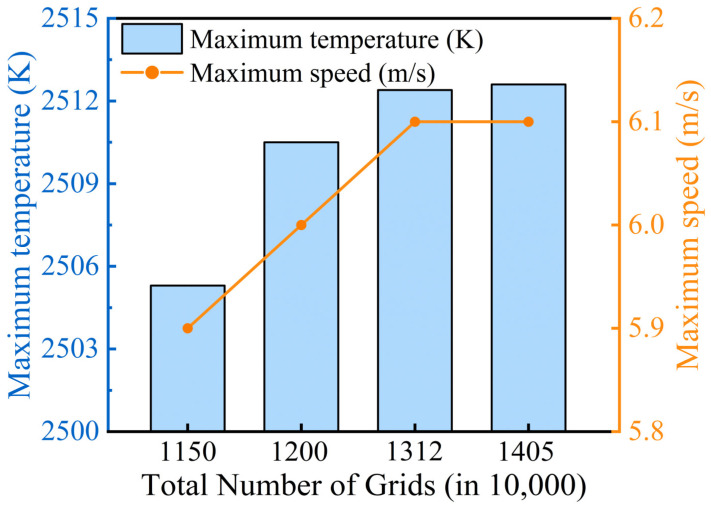
Grid independence verification results.

Due to the large size of the melting furnace model and the presence of irregular structures such as the neck, ports, and burner outlets, an unstructured mesh was generated using ANSYS 2022 R1 Meshing. The global mesh size was first controlled for the whole furnace, and local refinement was then applied to the burner outlets, port regions, neck connection, and high-temperature flame-development regions, where large gradients of velocity and temperature are expected. The mesh quality, orthogonal quality, and local refinement near key flow regions were used as criteria for mesh generation. To verify grid independence, four mesh schemes with total cell numbers of 11.5 million, 12.0 million, 13.12 million, and 14.05 million were compared. The maximum flame temperature and maximum flame velocity in the combustion space were selected as evaluation indicators because they are sensitive to the local flame structure, heat-release intensity, and jet-flow development. The relevant results are shown in Figure 4. When the number of cells increased from 11.5 million to 13.12 million, both maximum temperature and maximum velocity changed noticeably, indicating that the mesh was still affecting the calculation. When the number of cells increased further from 13.12 million to 14.05 million, the maximum velocity remained almost unchanged and the increase in maximum temperature became very small. Therefore, the mesh with 13.12 million cells was adopted for the subsequent comparative simulations to balance numerical accuracy and computational cost.

**Figure 5:**
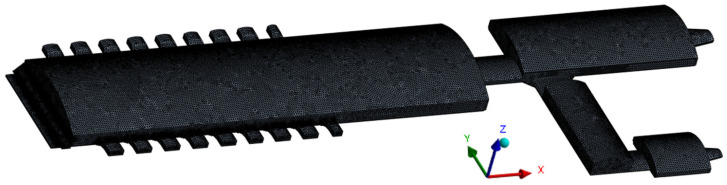
Grid division result of the overall melting furnace model.

After the grid independence verification, the overall grid of the float glass melting furnace is shown in Figure 5. The final mesh contains 13,127,407 cells and 2,262,638 nodes. The average element quality is 0.83, and the orthogonal quality is 0.86, indicating that the mesh quality meets the requirements of the present furnace-scale combustion-space simulation.

## 3. Model Description and Solution

To accurately simulate the combustion and heat transfer processes in the industrial float glass furnace, appropriate physical models and numerical solution methods were employed. This section introduces the turbulence, combustion, and radiation models adopted in the CFD simulations, together with the numerical solution procedures and convergence criteria used to ensure the stability and accuracy of the calculations.

### 3.1. Model Assumptions

To simplify the calculation and reduce the complexity of the simulation, the following assumptions were made for the model:

1. The combustion process is assumed to be steady, and the main flow, temperature, and species fields do not vary with time under each operating condition.
2. Because the gas velocity in the combustion space is relatively low, the gas flow is treated as a low-Mach-number flow. The density variation caused by temperature is described using the ideal-gas equation of state.
3. Natural gas and preheated air are introduced into the combustion space through separate inlets with prescribed mass-flow rates and uniform inlet profiles. After entering the furnace, they mix and react under the effects of turbulent jet interaction and furnace recirculation.
4. To reduce the complexity of the reaction system, the combustion chemistry was simplified by considering the main oxidation reactions of the hydrocarbon components in natural gas. Detailed intermediate reactions, pollutant-formation reactions, and chemical reactions of glass raw materials were not included.
5. In the gas-phase combustion space, turbulent convection and thermal radiation are regarded as the dominant heat-transfer mechanisms. Gas-phase molecular conduction is relatively weak at the furnace scale and is not treated as a dominant independent heat-transfer mode.

Although the present model captures the main gas-phase combustion, flow, convection, and radiation characteristics in the combustion space, these simplifications may influence the quantitative accuracy of local flame temperature, wall heat loss, radiative heat-transfer intensity, and species concentrations. Therefore, the present model is mainly suitable for comparing the relative effects of burner inclination angle and excess air ratio on the combustion-space temperature and flow fields, rather than for quantitatively predicting glass-melt flow, melting rate, glass quality, or pollutant emissions.

### 3.2. Solution of the Model

The numerical solution includes the governing equations for mass, momentum, energy, turbulence, species transport, combustion reaction, and radiative heat transfer. The Realizable k-ε model was used for turbulence, the Finite-Rate Model was used for natural-gas combustion, and the DO model was used for radiation. These models were selected to describe the coupled turbulent jet flow, furnace recirculation, chemical heat release, and high-temperature radiative heat transfer in the combustion space. The specific governing equations and model formulations are described below.

#### 3.2.1. Basic Equations of Computational Fluid Dynamics

When studying the temperature and flow fields of the flame, the mass, energy, and momentum conservation equations are the core mathematical tools for describing its physical nature. These three equations are coupled and work together to fully depict the flow behavior and heat transfer process of the fluid in the flame.

(1) Mass Conservation Equation

The conservation of mass equation describes the macroscopic transport behavior of gaseous mixtures involved in combustion in the furnace space. The equation is shown as follows:

(3) $$\frac{\partial \rho }{\partial t}+\frac{\partial (\rho u)}{\partial x}+\frac{\partial (\rho v)}{\partial y}+\frac{\partial (\rho w)}{\partial z}=0$$

where ρ is the density, kg·m^−3^; t is the time, s; and u, v, and w are the velocity components in the x, y, and z directions, respectively, m·s^−1^.

(2) Momentum Conservation Equation

The momentum conservation equation reflects the mechanical equilibrium in the fluid system and describes how the momentum of the fluid is distributed and evolves with time under the combined action of external forces, pressure, viscosity, and internal flow. The equation is described as follows [36].

(4) $$\frac{\partial \left(\rho \bar{v}\right)}{\partial t}+\nabla \left(\rho \bar{vv}\right)=-\nabla P+\nabla \cdot \left(\overset{̿}{\tau }\right)+\rho \vec{g}+\vec{F}$$

where P is the static pressure, Pa; $\overset{̿}{\tau }$ is the stress tensor; $\vec{g}$ is the gravitational acceleration vector, m·s^−2^; and $\vec{F}$ is the force source term per unit volume, N·m^−3^.

(3) Energy Conservation Equation

The energy conservation equation describes the thermal-energy changes in the melting furnace and provides a basis for optimizing the design of the melting furnace and improving energy efficiency. The energy equation is described as follows [30].

(5) $$\frac{\partial \left(\rho E\right)}{\partial t}+\nabla \cdot (\vec{v}(\rho E+P))=\nabla \cdot (k_{eff}\nabla T-\sum_{j}h_{j}{\vec{J}}_{j}+(\overset{̿}{\tau }eff\cdot \vec{v}))+S_{h}$$

where $k_{eff}$ is the effective thermal conductivity, W·m^−1^·K^−1^; ${\vec{J}}_{j}$ is the diffusion flux of component j; and the first three terms on the right-hand side represent the energy transfer due to conduction, mass diffusion, and viscous dissipation, respectively. $S_{h}$ is the heat of chemical reaction and other internal heat sources, KJ·m^−3^.

In the present model, the conduction term accounts for heat diffusion in the refractory solid domain and effective thermal diffusion in the gas phase. However, compared with turbulent convection and high-temperature radiation in the combustion space, gas-phase molecular conduction is not treated as a dominant heat-transfer mechanism.

#### 3.2.2. Turbulent Flow Model

The Realizable k–ε turbulence model was adopted to simulate the turbulent flow in the combustion space. Compared with the standard k–ε model, it provides improved predictions for flows involving strong jet interactions, large-scale recirculation, streamline curvature, which are characteristic of industrial float glass furnaces. In addition, the Realizable k–ε model offers a good balance between computational accuracy and efficiency and has been widely applied in CFD simulations of industrial combustion systems. The Realizable k-ε turbulence model consists of a turbulent kinetic energy equation and a dissipation-rate equation, as shown in Equations (6) and (7) [37]:

Turbulent kinetic energy equation:

(6) $$\frac{\partial }{\partial t}(\rho k)+\frac{\partial }{\partial xᵢ}(\rho kuᵢ)=\frac{\partial }{\partial xⱼ}\;[aₖ\;\mu_{\mathrm{eff}}\;\frac{\partial k}{\partial xⱼ}]+Gₖ+G_{b}-\rho \varepsilon +Sₖ$$

where k is the turbulent kinetic energy, m^2^·s^−2^; $u_{i}$ is the velocity component in the $x_{i}$ direction, m/s; $\alpha_{k}$ diffusion coefficient corresponding to turbulent kinetic energy; $\mu_{\mathrm{eff}}$ is the effective dynamic viscosity, $\mu_{\mathrm{eff}}$ = $\mu +\mu_{t}$, where μ is the molecular dynamic viscosity, $\mu_{t}$ is the turbulent dynamic viscosity, Pa·s; $G_{k}$ is the turbulent kinetic energy generated by the average velocity gradient, kg·m^−1^·s^−3^; $G_{b}$ is the generation of turbulent kinetic energy due to buoyancy, J·m^−3^·s^−1^; ε is the turbulent dissipation rate, m^2^·s^−3^; and $S_{k}$ is the source term for turbulent kinetic energy, kg·m^−1^·s^−3^.

Dissipation rate equation:

(7) $$\frac{\partial (\rho \varepsilon )}{\partial t}+\frac{\partial }{\partial xⱼ}(\rho \varepsilon uⱼ)=\frac{\partial }{\partial xⱼ}[(\mu +\frac{\mu ₜ}{\sigma_{\varepsilon }})\frac{\partial k}{\partial xⱼ}]+\rho C_{1}E\varepsilon -C_{2}\rho \frac{\varepsilon^{2}}{k+\surd (\varepsilon \nu )}+C_{1\varepsilon }\frac{\varepsilon }{k}C_{3\varepsilon }G_{b}+S_{\varepsilon }$$

where $\sigma_{\varepsilon }$ is the turbulent Prandtl number for the dissipation rate; v is the kinematic viscosity, m^2^/s; $C_{1},{\;C}_{2},{\;C}_{1\varepsilon },{\;C}_{3\varepsilon }$ are the model constants; and $S_{\varepsilon }$ is the source term of the dissipation rate, kg·m^−1^·s^−3^.

The initial kinetic energy and initial kinetic energy dissipation rate at the inlet are determined by Equations (8) and (9) [38]:

(8) $$K_{inlet}=0.01v_{inlet}^{2}$$

(9) $$\varepsilon_{inlet}=0.01v_{inlet}^{2}\frac{{2k}_{inlet}^{1.5}}{D_{inlet}}$$

where $K_{inlet}$ is the initial kinetic energy, m^2^·s^−2^; $v_{inlet}$ is the average flow velocity at the inlet, m/s; $\varepsilon_{inlet}$ is the initial turbulent dissipation rate, m^2^·s^−3^; and $D_{inlet}$ is the inlet characteristic length, m.

#### 3.2.3. Combustion Reaction Model

Combustion in the float glass melting furnace was modeled using the Species Transport model coupled with the Finite-Rate/Eddy-Dissipation combustion model. Since natural gas and preheated combustion air enter the furnace through separate inlets and mix within the combustion space, the combustion process can be regarded as turbulent non-premixed combustion. In the Finite-Rate/Eddy-Dissipation model, the local reaction rate is determined by both Arrhenius chemical kinetics and turbulent mixing, with the overall reaction rate controlled by the limiting mechanism. This model is well suited for industrial glass furnaces, where combustion is governed by the combined effects of finite-rate chemistry and intense turbulent mixing generated by high-velocity burner jets.

(10) $${CH}_{4}+{2O}_{2}\to {CO}_{2}+{2H}_{2}O$$

(11) $$C_{2}H_{6}+\frac{7}{2}O_{2}\to {2CO}_{2}+{3H}_{2}O$$

The finite-rate reaction rates were calculated using the Arrhenius formulation (12):

(12) $$k=AT^{n}exp(-\frac{E_{a}}{RT})$$

where k is the reaction rate constant, A is the pre-exponential factor, T is the absolute temperature (K), n is the temperature exponent, $E_{a}$ is the activation energy, and R is the universal gas constant.

#### 3.2.4. The DO Model

Due to the high temperature inside the float glass melting furnace, radiative heat transfer plays a crucial role in the overall heat-transfer process. The Discrete Ordinates (DO) Model was adopted since it is applicable to radiative transfer in complex 3D furnace geometries, and capable of accounting for radiative exchange among flue gas, refractory walls and molten glass surfaces. The Radiative Transfer Equation (RTE) describing this radiative heat transfer process in the space is as follows [39]:

(13) $$div\left(I\left(\vec{r},\vec{s}\right)\vec{s}\right)+\left(a+\sigma_{s}\right)I\left(\vec{r},\vec{s}\right)=an^{2}\cdot \frac{\sigma T^{4}}{\pi }+\frac{\sigma_{s}}{4\pi }\int_{0}^{4\pi }I(\vec{r},\vec{s}\prime )\phi (\vec{s},\vec{s}\prime )d\Omega$$

where $\vec{r}$, $\vec{s}$, $\vec{s}\prime$ respectively for the position vector; direction vector, and scattering direction vector; s is the path length, m; *a*, *n*, *σ_s_* respectively for the absorption index, refractive index, and heat dissipation coefficient; *I* is the radiation intensity affected by $\vec{r}$ and $\;\vec{s}$, W·m^−2^·sr^−1^; *T* is the local temperature, K; ϕ is the phase function; and Ω is the solid angle, sr.

#### 3.2.5. Solution Method

After comprehensively considering the turbulence model, conservation equations, combustion model, and radiation model, the coupled algorithm was selected for pressure–velocity coupling. In the present furnace-scale calculation, first-order upwind discretization was used to improve numerical stability and convergence robustness. It should be noted that first-order discretization may introduce numerical diffusion and smooth sharp gradients of temperature and velocity. Therefore, the present results are mainly used for relative comparison among different operating conditions. For future high-precision prediction of local flame structure, wall heat flux, and pollutant formation, second-order or higher-order discretization schemes should be adopted and further verified. The root mean square (SRSS) method was adopted as the calculation convergence criterion. The residual convergence standard for the energy equation was set to no more than 1 × 10^−6^, and that for the other equations was set to no more than 1 × 10^−3^.

### 3.3. Parameter Selection and Model Applicability

The investigated burner inclination angles of 5°, 10°, and 15° were selected according to the practical installation range of under-port natural gas burners in the studied furnace. This range covers the commonly used small-angle injection mode and allows the influence of jet direction on flame spreading and flue gas recirculation to be compared. The excess air ratios of 1.0, 1.05, 1.10, 1.15, and 1.20 were selected to cover the transition from near-stoichiometric combustion to moderately air-rich combustion. In industrial operation, an excess air ratio equal to 1.0 means that the supplied air equals the theoretical air demand, but complete combustion is usually difficult because of imperfect mixing and finite reaction time. Therefore, a moderate excess air ratio is commonly required to improve combustion completeness, while too much excess air may increase heat loss carried by flue gas. The selected parameter range is thus representative of practical operating adjustment for the present natural gas-fired float glass furnace.

## 4. Model Validation and Simulation Result Analysis

This section evaluates the reliability of the developed CFD model through comparison with experimental measurements. After the model validation, the effects of burner inclination angle and excess air ratio on the combustion characteristics, temperature distribution, flow field, and heat transfer performance of the industrial float glass furnace are systematically analyzed.

### 4.1. Model Validation

**Figure 6:**
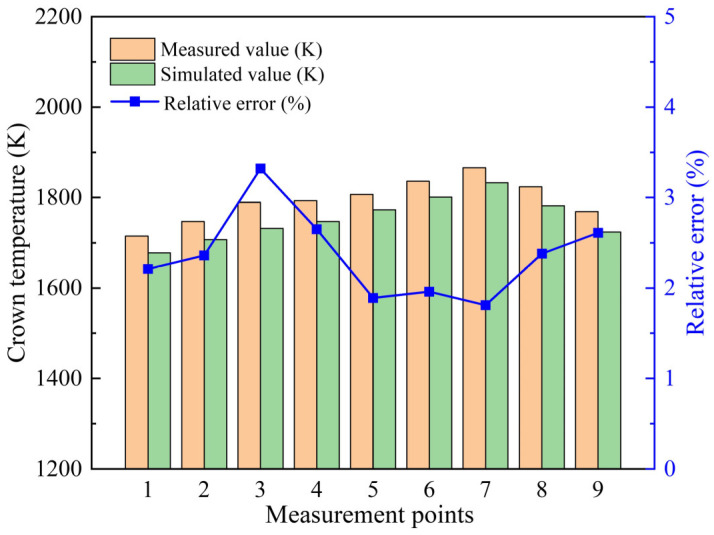
Relative errors between numerical and experimental results.

The accuracy of the model is a key prerequisite to ensure that the simulation results are instructive. To verify whether the established glass melting furnace simulation model can truly reflect the actual thermal state of the furnace, this study selects nine typical positions on the crown of a 1300 t/d glass melting furnace and conducts temperature measurement experiments under standard working conditions. The measurement results are compared with the simulation calculation values to verify the reliability of the model. The results are shown in Figure 6.

It reveals that the variation trends of the simulated values and the measured values are basically consistent. The simulated values are all slightly lower than the measured values, which is because the model is simplified compared to the actual equipment, and the heat transfer and combustion chemical reactions are not as complex as in the actual process. By comparing the simulated and measured values, the maximum relative error between the two is 3.3%, and the corresponding temperature difference is 57 K. Since the maximum error is less than 5%, the model can meet the needs of the research.

### 4.2. Analysis of Simulation Results

**Figure 7:**
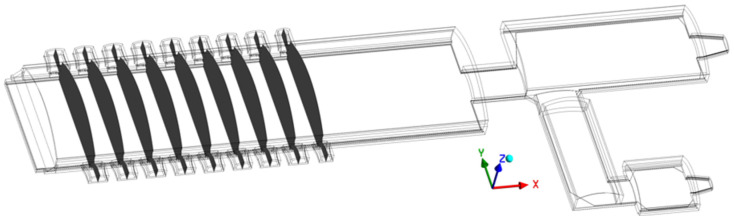
Schematic diagram of the central cross-sections positions of each port.

During combustion, natural gas is injected into the combustion space through the burners under the ports, while preheated air enters through the corresponding air inlets. The fuel jet interacts with the air stream and the recirculating flue gas in the furnace, forming turbulent mixing and combustion regions. Therefore, the flame length, high-temperature radiation region, and heat-release distribution are affected by burner inclination angle and the mass flow rates of air and natural gas. This study mainly analyzes the temperature and flow distribution of the central cross-sections of the nine ports under different burner installation angles β and different excess air ratios α. The positions of these cross-sections are shown in Figure 7. Schematic diagram of central cross-sections positions of each port. Based on the temperature and flow-field results, the effects of burner angle and excess air ratio on the combustion-space thermal environment were further analyzed.

#### 4.2.1. Influence of Burner Angle on Combustion Process

The installation inclination angle of the natural gas burner in a natural gas-fired float glass-melting furnace is denoted as β. It is defined as the angle between the burner centerline and the horizontal direction of the glass melt surface, as shown in Figure 3c. A suitable range is 5–15°. This paper mainly studies the cases where the installation inclination angle β of the burner is 5°, 10°, and 15°.

(1) Influence on the Combustion-Temperature Distribution

**Figure 8:**
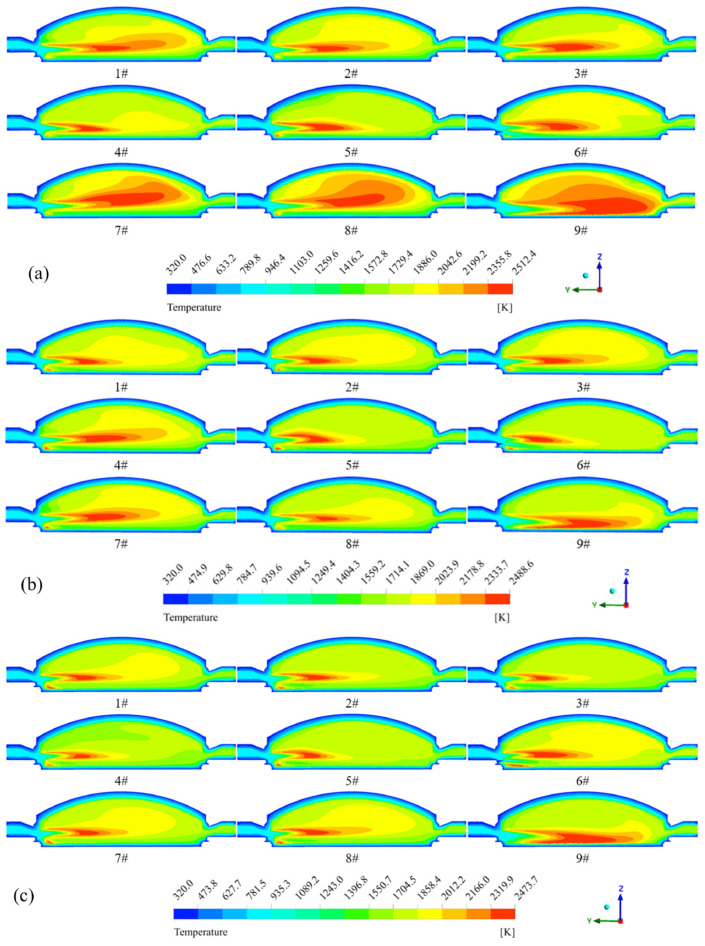
Temperature distribution of the central cross-sections of each port: (**a**) β = 5°; (**b**) β = 10°; and (**c**) β = 15°.

Under different burner inclination angles, the temperature distribution of the central cross-sections corresponding to each port is presented in Figure 8. The temperature distribution and the maximum temperature values at the nine ports were compared for β = 5°, 10°, and 15°. When the burner angle increases from 5° to 15°, the flame length gradually decreases, and the range of the high-temperature region also decreases. This indicates that when β = 5°, the natural gas jet is distributed more horizontally in the combustion space and has a larger contact area with the preheated air stream inside the furnace, promoting turbulent mixing and combustion. The maximum temperature at β = 5° is 2512 K, which is 24 K and 39 K higher than those at β = 10° and β = 15°, respectively. This suggests that when the inclination angle is smaller, the flame can form a wider high-temperature region under the present operating conditions.

**Figure 9:**
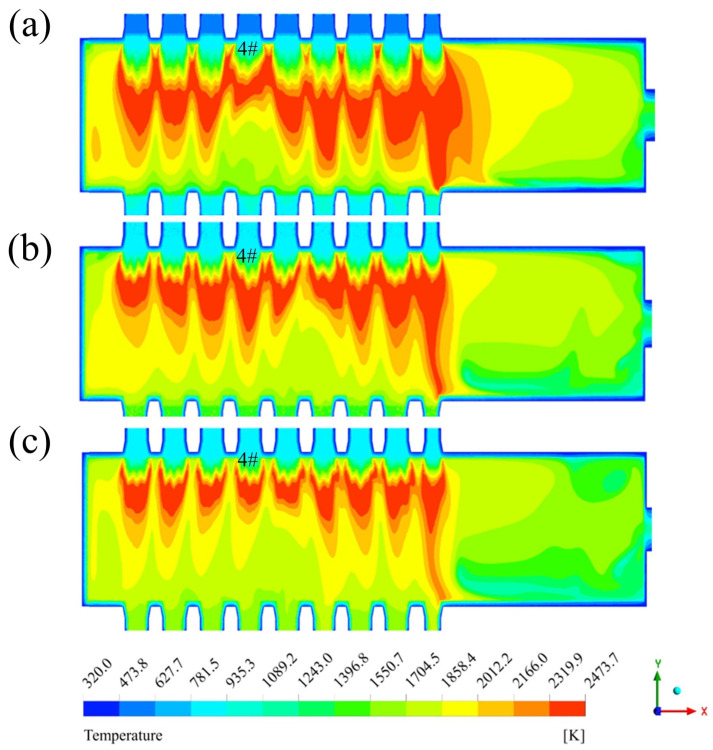
Temperature distribution of the cross-sections along the height direction (Z direction) of the melting furnace: (**a**) β = 5°, Z = 0.35 m; (**b**) β = 10°, Z = 0.45 m; and (**c**) β = 15°, Z = 0.53 m.

For different burner angles, Figure 9 depicts the temperature distribution of the melting section along the height direction. Each of the nine ports generates a corresponding flame region. According to the distribution results, the temperature distribution in the Z direction corresponds to the temperature distribution of the central cross-section in Figure 8. As the burner angle increases, the flame length, flame width, and high-temperature radiation region decrease, whereas the ignition distance increases. This occurs because a smaller inclination angle allows the fuel jet to spread more effectively in the near-burner region and to interact earlier with preheated air and high-temperature recirculating flue gas. As a result, the ignition region appears closer to the front wall, and the combustion process becomes more stable.

In addition, the leftward inclination angle of the flame of the 4# port decreases with the increase in the burner inclination angle, while the flame length increases instead. This is because the gas flow rates of the first three ports are relatively large, and the surrounding gas is rapidly heated, which affects the flow direction of the flame of the 4# port, resulting in inclination.

(2) Influence on the Combustion Flow Distribution

**Figure 10:**
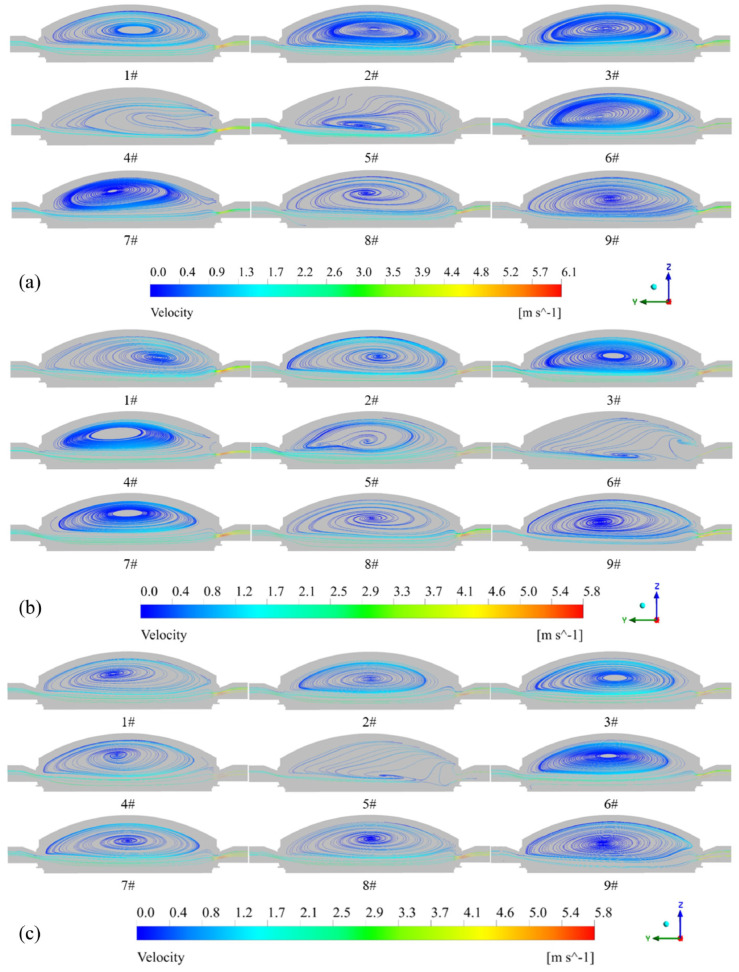
Streamline distribution of the central cross-sections of the ports: (**a**) β = 5°; (**b**) β = 10°; and (**c**) β = 15°.

Figure 10 displays the velocity streamline distribution of the central cross-sections of each port under different burner inclination angles. The flue gas flow distribution is related to the temperature distribution of the central cross-sections of each port. When β = 5°, a large amount of flue gas accumulates above the flame jets of most ports, but the flue gas above the 4# and 5# ports become sparse due to the interference of other high-temperature gas flows. When the burner angle is 10°, the amount of backflow flue gas above the flame jets of the ports decreases. The 5# port is slightly affected by gas flow interference, and the flue gas above the 6# port is severely interfered with by the adjacent gas flow, resulting in only a small amount of flue gas backflow. When the burner angle is 15°, the amount of backflow flue gas above the flame jets of the ports becomes even less, but the distribution is relatively uniform. The reason is that when the inclination angle is smaller, the flame is more concentrated, which increases the local temperature difference, leading to greater gas flow fluctuation effects on some ports and uneven distribution of flue gas backflow. Although there is local unevenness in the heat transfer process, the overall heat transfer amount and efficiency are relatively large. When the angle increases, the heat transfer uniformity of the flue gas is better, but the heat transfer amount and heat transfer efficiency are lower. Therefore, it is considered that the melting effect of the burner with an inclination angle of 5° is better than that of 15°.

**Figure 11:**
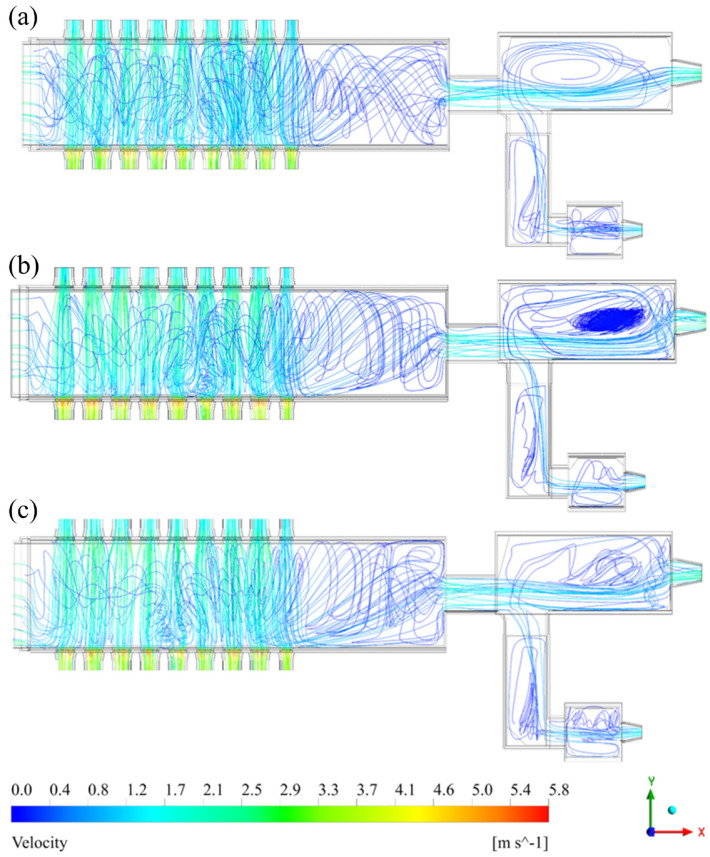
Streamline distribution of the entire melting furnace: (**a**) β = 5°; (**b**) β = 10°; (**c**) β = 15°.

Under different burner inclination angles, the velocity streamline distribution across the entire melting furnace is illustrated in Figure 11. When β = 5°, the longitudinal and transverse backflow of the flue gas in the melting part is more intensive, but that in the cooling part is relatively sparse. The opposite trend is observed at 10° and 15°. The reason is that when the burner inclination angle is smaller, the flame generated in the melting part is more concentrated, the temperature is higher, and the flue gas backflow is more intense. This is consistent with the conclusion in the literature [40] that more obvious lateral and longitudinal backflow can be generated after the combustion of preheated flue gas. The temperature difference between the cooling part and the melting part is huge, and a large amount of flue gas is discharged from the ports or flows back to the melting part, resulting in only a small amount of flue gas reaching the cooling part.

In conclusion, the burner inclination angle changes the jet direction and momentum distribution, thereby affecting the interaction among jet flow, furnace recirculation, and buoyancy-driven upward motion of high-temperature flue gas. The observed variation in flame length, ignition distance, and high-temperature region is therefore the combined result of jet direction, turbulent mixing, recirculation, and buoyancy. In the present model, buoyancy is included through the gravity term and temperature-dependent gas density, but its isolated contribution to flame morphology was not quantified separately.

#### 4.2.2. Influence of Excess Air Ratio α on the Combustion Process

In industrial practice, the air input has an important influence on natural gas combustion. When the excess air ratio α is less than 1, it leads to low combustion efficiency and higher pollutant emissions. When α = 1.0, the supplied air is equal to the theoretical air demand. When α is greater than 1, the combustion is more complete, but the excess air will take away part of the heat, leading to a temperature drop and thus affecting the combustion efficiency. Therefore, it is important to study the influence of the excess air ratio on the combustion of the ports in the glass melting furnace. This study investigated the influence of the excess air ratio α on the combustion process when α is 1.0, 1.05, 1.10, 1.15, and 1.20. The temperature distributions of the central cross-sections of the 3# and 8# ports were mainly analyzed. Port 3# is located in the central area of the batch melting, and port 8# is located in the hot spot central area. Both have a great influence on the melting part.

(1) Influence on the Combustion-Temperature Distribution

**Figure 12:**
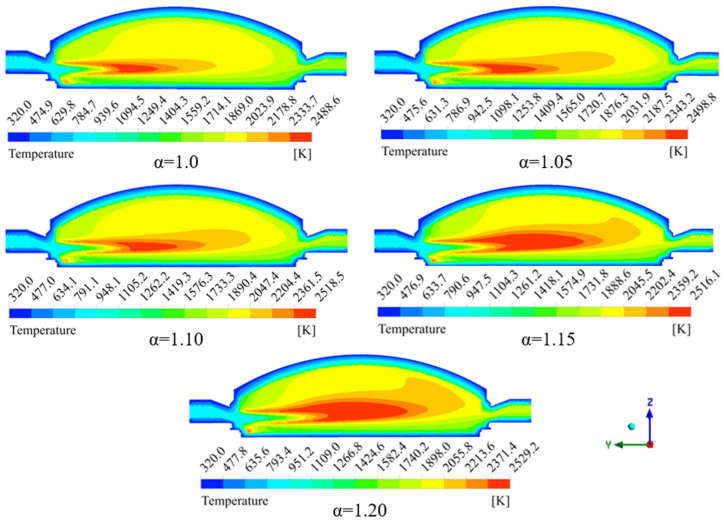
Temperature distribution of the central cross-section of port 3# under different excess air ratios.

**Figure 13:**
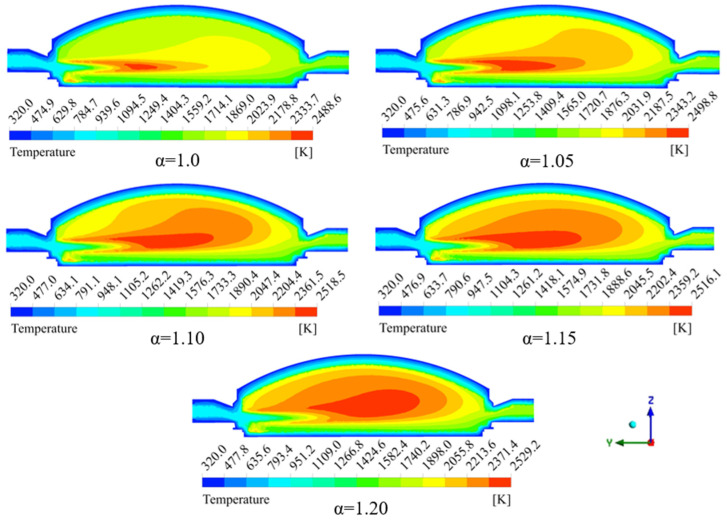
Temperature distribution of the central cross-section of port 8# under different excess air ratios.

Figure 12 and Figure 13 are the temperature distributions of the central cross-section of ports 3# and 8# under different excess air ratios, respectively. With the increase in the excess air ratio, the flame temperature increases, the flame length becomes longer, and the high-temperature region becomes wider in both figures. The maximum temperature increases from the initial 2489 K to 2529 K, an increase of 40 K. However, when the excess air ratio increases to 1.15, compared with the working condition when the excess air ratio is 1.10, the flame temperature decreases slightly, and the increasing trend of the flame radiation region is almost unaffected.

The variation law and trend of the flame of port 8# are basically consistent with those of port 3#. When the excess air ratio increases to 1.20, compared with when α is 1.15, the increasing trends of the temperature and radiation region are very small. However, under this situation, due to the relatively large gas flow rate, the natural gas combustion is very intense, the ignition point of the flame is relatively far away, and the combustion area is relatively large. If the excess air ratio is too large, the remaining air after combustion will take away the heat around the flame, and at this time, the temperature around the flame will no longer increase or even decrease slightly.

**Figure 14:**
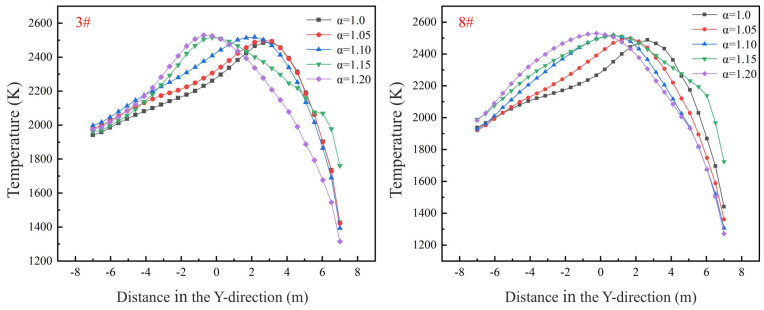
Temperature variation curves along the centerline of ports 3# and 8# under different excess air ratios.

To further observe the temperature changes, Figure 14 displays the variation curves of the temperature along the centerline of ports 3# and 8# with the furnace width Y direction under different excess air ratios, where Y = 0 is located at the center of the melting part of the float glass melting furnace. This figure corresponds to the variation law of the flame shown in Figure 11 and Figure 12. The flame lengths of both increase with the increase in the excess air ratio. When the excess air ratio α increases from 1.0 to 1.20, both flame length and maximum flame temperature gradually increase. When α increases from 1.10 to 1.15, the increasing trends of the flame length and the range of the high-temperature region are the most obvious. However, when α increases from 1.15 to 1.20, the flame length, the temperature, and the high-temperature region of the flame basically do not change; the combustion performances of both are basically stable.

When the excess air ratio increases to 1.20, the maximum temperature and the high-temperature region no longer increase significantly. Under this condition, additional excess air may carry away more sensible heat with the flue gas. Therefore, within the selected parameter range and under the present combustion-space model, α = 1.15 is regarded as a preferred value rather than an absolute global optimum. When the excess air ratio is between 1.10 and 1.15, the combustion-space thermal condition can be improved without introducing excessive air-related heat loss.

(2) Influence of Excess Air Ratio α on the Combustion Flow-Field Distribution

**Figure 15:**
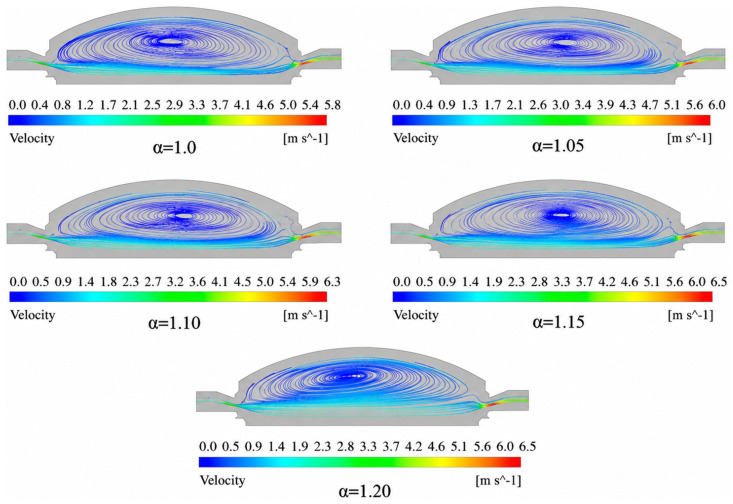
Streamline distribution plots of the central cross-section of port 3# under different excess air ratios.

**Figure 16:**
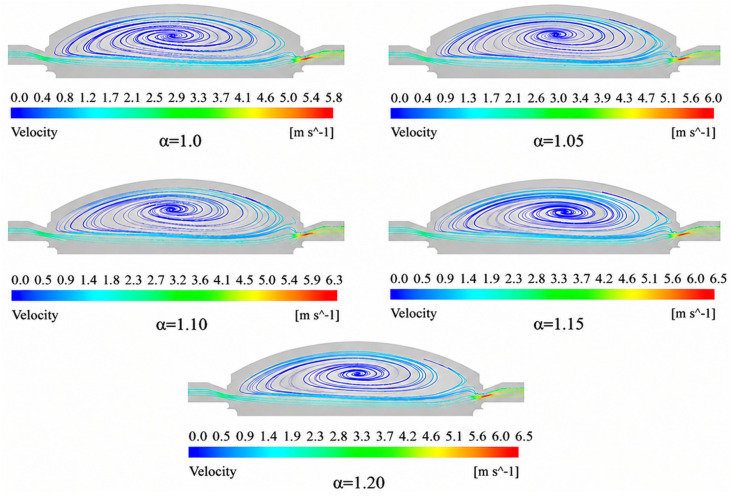
Streamline distribution plots of the central cross-section of port 8# under different excess air ratios.

Figure 15 and Figure 16 are the streamline distributions of the central cross-section of ports 3# and 8# under different excess air ratios. Figure 14 displays that with the increase in the excess air ratio, the amount of longitudinally backflowing flue gas decreases, but the velocity of the flue gas gradually increases. Moreover, the flue gas accumulates on the left wall and the arch top. The reason is that when α increases, the combustion becomes more complete, and the generated jet flame is longer. When approaching the right exhaust furnace port, the high-velocity flue gas region is relatively large, squeezing the backflow flue gas to the left for accumulation.

Since port 8# is located in the hot spot region, combustion is more complete, and the amount of recirculating flue gas is relatively small. It can be clearly seen that the number of streamlines is much less than that of port 3#. Moreover, with the increase in α, the amount of accumulated flue gas gradually decreases.

**Figure 17:**
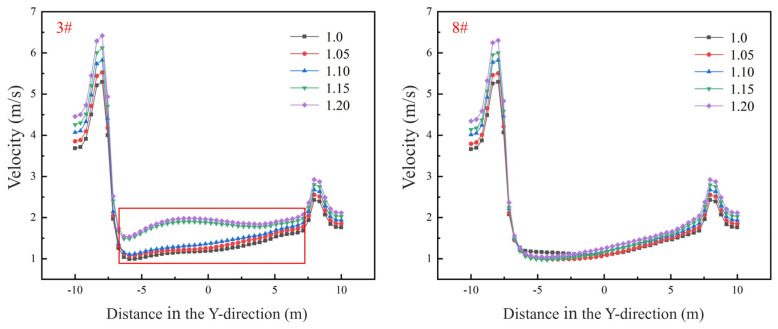
Flow velocity variation curves along the centerline of ports 3# and 8# under different excess air ratios.

Figure 17 shows the variation curves of the flow velocity along the centerline of ports 3# and 8# with the furnace width Y direction under different excess air ratios.

The sudden rise in the curve is due to the abrupt narrowing of the nozzle outlet cross-section relative to the dimensions of the combustion space, which results in an acceleration of the flow. When α is 1.15 and 1.20, the flow velocity in the flame combustion region increases significantly, which can be clearly seen in the red box part of the figure. The reason is that when a sufficient amount of air comes into contact with natural gas and burns rapidly, the generated flue gas can be discharged quickly, so the flow velocity is higher than that of when the excess air ratio is smaller.

However, the increase in velocity along the centerline of port 8# is very small, and several curves almost overlap. In the hot-spot region, the flame distribution is relatively uniform, and the interaction between gas flows is small. With the increase of α, the flue gas flow velocity at different positions does not change significantly.

**Figure 18:**
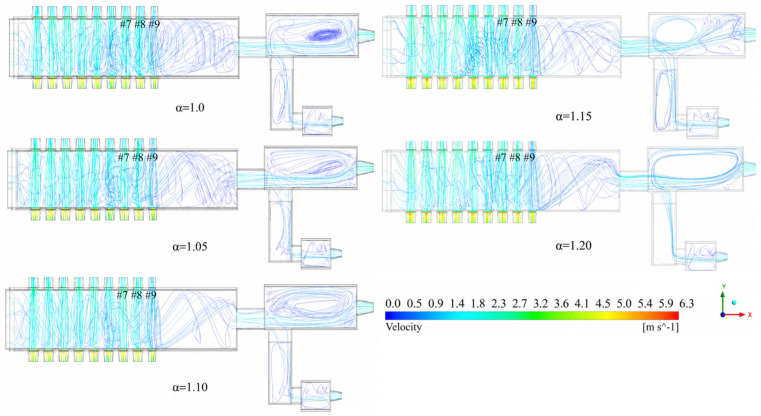
Streamline distribution of the entire melting furnace under different excess air ratios.

The streamline distribution of the entire melting furnace under different excess air ratios is shown in Figure 18. With the increase of α, the overall longitudinal and transverse backflow of the flue gas shows a decreasing trend, but the longitudinal and transverse backflow at ports 7#, 8#, and 9# does not decrease, while the flue gas backflow at the cooling part, the end of the melting part, and the first few ports decreases. The reason is that after more air enters, the driving force of the flue gas backflow formed by the temperature and pressure difference is weakened, and the overall longitudinal and transverse backflow is inhibited, showing a decreasing trend. At the same time, the excess air will “dilute” the combustion heat, making the overall temperature field in the furnace more uniform, and the natural flue gas backflow formed by the local high and low temperature difference is weakened, leading to a reduction in the large-scale backflow.

**Figure 19:**
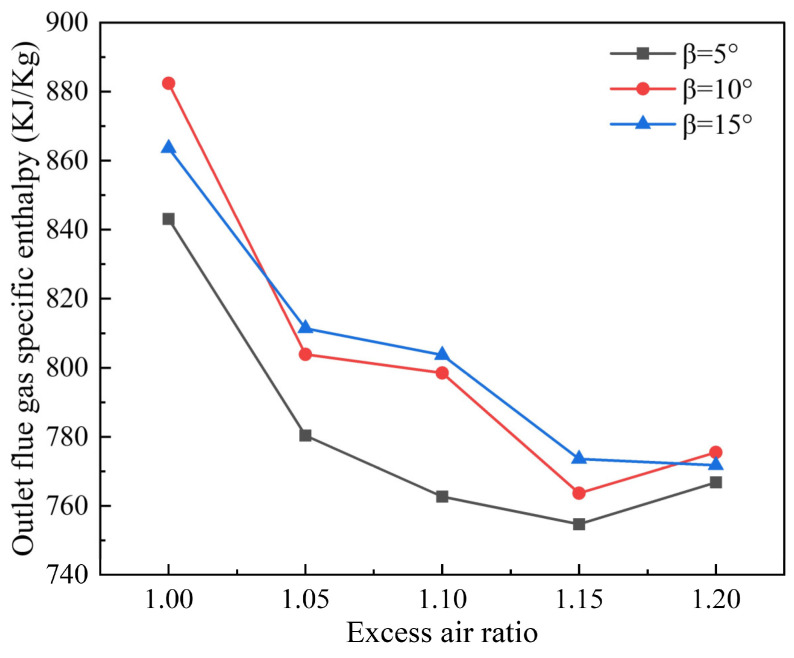
Effect of burner inclination angle and excess air ratio on outlet flue-gas-specific enthalpy.

To further verify the optimum operating conditions, the outlet flue-gas specific enthalpy was employed as a quantitative indicator to evaluate the sensible heat loss carried by the exhaust gas. A lower outlet flue gas enthalpy indicates that less thermal energy is discharged from the furnace with the flue gas, implying more efficient utilization of the released combustion heat. The variations in outlet flue-gas specific enthalpy under different burner inclination angles and excess air ratios are presented in Figure 19. β = 5° and α = 1.15 achieve the lowest outlet flue-gas specific enthalpy of 755 KJ/Kg.

## 5. Conclusions

This study developed a three-dimensional CFD model of the combustion space of a 1300 t/d natural gas-fired float glass furnace with a single-furnace dual-cooler configuration. The model was validated against crown-temperature measurements, and the maximum relative error was 3.3%, indicating that it is suitable for comparative analysis of combustion-space temperature and flow fields. The results show that the dual-cooler layout and neck region alter the flue-gas flow path, making flame development and heat distribution sensitive to both burner inclination angle and excess air ratio.

For the burner inclination angle, reducing β from 15° to 5° enhanced the horizontal extension of the natural-gas jet and increased its contact area with preheated air. At β = 5°, a wider high-temperature region was formed, and the maximum flame temperature reached 2512 K, which was 24 K and 39 K higher than those at β = 10° and β = 15°, respectively. Although larger angles improved local flow uniformity, they shortened the flame and weakened the high-temperature radiation region. Therefore, β = 5° is preferred within the investigated range.

For the excess air ratio, increasing α from 1.0 to 1.15 promoted combustion completeness, extended the flame region, and strengthened the high-temperature zone. However, increasing α further to 1.20 produced only limited improvement and may increase sensible heat loss through excess flue gas. Thus, α = 1.15 is preferred under the present operating conditions.

Overall, the combined condition of β = 5° and α = 1.15 provides a more favorable combustion-space thermal environment for this single-furnace dual-cooler float glass furnace. These findings provide guidance for burner arrangement and air-fuel-ratio adjustment in similar industrial furnaces. Future work should further couple the combustion-space model with glass-melt flow, batch melting, pollutant formation, and higher-order discretization schemes to improve quantitative prediction accuracy.

## Figures and Tables

**Table 1:** Fuel distribution ratio.

**NO.**	**1#**	**2#**	**3#**	**4#**	**5#**	**6#**	**7#**	**8#**	**9#**
Fuel ratio/%	11	15	15	15	11	8	14	8	3
Number of burners	4	4	4	4	4	3	4	4	2

**Table 2:** Summary of main boundary conditions.

**Category**	**Boundary Item**	**Specific Parameter Value and Description**
Fuel inlet	Mass-flow inlet	Natural gas, T = 300 K, 2.3 kg/s
Preheated air inlet	Mass-flow inlet	Regenerator preheat T = 665 K, O_2_ mass fraction 21%,10.5 kg/s
Flue gas outlet	Pressure outlet	Gauge pressure = 0 Pa, backflow T = 1350 K
Inner furnace	Wall	Emissivity = 0.6
Outer walls of the furnace	Wall	h = 5 W/(m^2^·K), Emissivity = 0.6 T atmosphere = 320 K
Glass-melt surface	Wall	Emissivity = 0.75

**Table 3:** Physical parameters of each component and material.

**Materials**	**Density** **(kg/m^3^)**	**Thermal Conductivity** **(W·m^−1^·K^−1^)**	**Viscosity** **(kg·m^−1^·s^−1^)**	**Heat Capacity** **(J·kg^−1^·K^−1^)**
Refractory Brick	2600	1.6	-	1250
CH_4_	0.6679	0.0332	1.087 × 10^−5^	Refer to the Appendix A
C_2_H_6_	1.263	0.0207	9.29 × 10^−6^	
CO_2_	1.7878	0.0145	1.37 × 10^−5^	
H_2_O	0.5542	0.0261	1.34 × 10^−5^	
Air	0.263	0.0246	1.919 × 10^−5^	

## Data Availability

The original contributions presented in this study are included in the article. Further inquiries can be directed to the corresponding author.

## Appendix A

**Table A1:** Relationships between the constant-pressure specific heat capacity and temperature for each material in Table 3.

Materials	Temperature Range	$C_{p}$ Calculation Formula
CH_4_	298 K–1300 K	$C_{p}=223.5+346.8*10^{-3}T+\left(-135.6\right)*10^{-6}T^{2}+18.7*10^{-9}T^{3}+{218.5*10}^{3}T^{-2}$
	1300 K–6000 K	$C_{p}=660.8+79.4*10^{-3}T+\left(-14.8\right)*10^{-6}T^{2}+1.1*10^{-9}T^{3}+{(-1162.5)*10}^{3}T^{-2}$
C_2_H_6_	298 K–1300 K	$C_{p}=385.2+528.6*10^{-3}T+\left(-216.3\right)*10^{-6}T^{2}+32.4*10^{-9}T^{3}+{382.7*10}^{3}T^{-2}$
	1300 K–6000 K	$C_{p}=986.5+142.3*10^{-3}T+\left(-31.7\right)*10^{-6}T^{2}+2.5*10^{-9}T^{3}+{(-2156.8)*10}^{3}T^{-2}$
CO_2_	298 K–1300 K	$C_{p}=442.1+87.5*10^{-3}T+\left(-64.3\right)*10^{-6}T^{2}+24.1*10^{-9}T^{3}+{(-86.2)*10}^{3}T^{-2}$
	1300 K–6000 K	$C_{p}=658.3+28.9*10^{-3}T+\left(-1.2\right)*10^{-6}T^{2}+0.3*10^{-9}T^{3}+{(-1684.5)*10}^{3}T^{-2}$
H_2_O	298 K–1300 K	$C_{p}=1405.8+143.2*10^{-3}T+\left(-48.6\right)*10^{-6}T^{2}+8.6*10^{-9}T^{3}+{(-254.3)*10}^{3}T^{-2}$
	1300 K–6000 K	$C_{p}=1782.4+62.5*10^{-3}T+\left(-8.9\right)*10^{-6}T^{2}+0.7*10^{-9}T^{3}+{(-2038.6)*10}^{3}T^{-2}$
Air	298 K–1300 K	$C_{p}=910.2+158.3*10^{-3}T+\left(-71.6\right)*10^{-6}T^{2}+14.3*10^{-9}T^{3}+{(-201.5)*10}^{3}T^{-2}$
	1300 K–6000 K	$C_{p}=1056.8+76.4*10^{-3}T+\left(-15.2\right)*10^{-6}T^{2}+1.2*10^{-9}T^{3}+{(-1892.3)*10}^{3}T^{-2}$
